# Evaluating the model of offering expanded genetic carrier screening to high school students within the Sydney Jewish community

**DOI:** 10.1007/s12687-021-00567-8

**Published:** 2021-11-30

**Authors:** Kristine Barlow-Stewart, Kayley Bardsley, Elle Elan, Jane Fleming, Yemima Berman, Ron Fleischer, Krista Recsei, Daniel Goldberg, John Tucker, Leslie Burnett

**Affiliations:** 1 Northern Clinical School, Faculty of Medicine and Health, University of Sydney, St Leonards, NSW 2065 Australia; 2 Community Genetics Program (NSW), Wolper Jewish Hospital, Woollahra, NSW 2025 Australia; 3 Department of Genetic Medicine, Westmead Hospital, Westmead, NSW 2145 Australia; 4 Faculty of Health, University of Technology Sydney, Ultimo, NSW 2007 Australia; 5 Department of Clinical Genetics, Royal North Shore Hospital, St Leonards, NSW 2065 Australia; 6 Department of Medical Genomics, Royal Prince Alfred Hospital, Camperdown, NSW 2010 Australia; 7 Pangolin Consulting, The Entrance, NSW 2261 Australia; 8 Kinghorn Centre for Clinical Genomics, Garvan Institute of Medical Research, Darlinghurst, NSW 2010 Australia; 9 St Vincent’s Clinical School, UNSW Sydney, Darlinghurst, NSW 2010 Australia

**Keywords:** Pre-conception reproductive carrier testing, Expanded genetic carrier testing, Genetics education, High school screening program, Ashkenazi Jewish community genetics screening

## Abstract

Programs offering reproductive genetic carrier screening (RGCS) to high school students within the Ashkenazi Jewish community in several countries including Canada and Australia have demonstrated high uptake and retention of educational messages over time. This study was undertaken to evaluate whether testing for an expanded number of conditions in a high school setting would impact the effectiveness of education. In this questionnaire-based study, genetic carrier testing for nine conditions was offered to 322 year 11 students from five high schools, with students attending a compulsory 1-h education session prior to voluntary testing. Comparison of pre- and post-education measures demonstrated a significant increase in knowledge, positive attitudes, and reduced concern immediately after the education session. Retention of knowledge, measures of positive attitude, and low concern over a 12-month period were significantly higher than baseline, although there was some reduction over time. In total, 77% of students exhibited informed choice regarding their intention to test. A significant increase in baseline knowledge scores and positive attitude was also demonstrated between our original 1995 evaluation (with testing for only one condition) and 2014 (testing for nine conditions) suggesting community awareness and attitudes to RGCS have increased. These findings validate the implementation of effective education programs as a key component of RGCS and are relevant as gene panels expand with the introduction of genomic technologies.

**Supplementary Information:**

The online version contains supplementary material available at 10.1007/s12687-021-00567-8.

## Introduction

Developments enabling fast and cost-effective genomic technologies have led to the increased availability of reproductive genetic carrier screening (RGCS) for conditions that follow a pattern of autosomal recessive (AR) inheritance (Hallam et al. 2014; Lazarin et al. 2013; Rose and Wick 2018). When both biological parents are genetic carriers for the same condition, there is a one in four chance in each pregnancy of having an affected child.

Reproductive choices are enabled when parental genetic carrier status is known. These choices are further increased if the information is known pre-conception as they enable carriers to choose to utilise preimplantation genetic testing or donor gametes, access early pregnancy diagnostic testing, or not have biological children. For this reason, offering RGCS in the pre-conception period is considered preferable to early pregnancy (Edwards et al. 2015; Henneman et al. 2016). There is however no consensus as to the preferred model for offering RGCS: to couples or individuals (Janssens et al. 2017). For public health programs, the former provides the greatest benefit in terms of reduction of workload in returning results as there would be far fewer carrier couples identified (Kraft et al. 2019). Indeed, a number of studies underway internationally are investigating how best to offer RGCS to couples, for example, the Australian Genomics Health Alliance (Australian Genomics) study Mackenzie’s Mission (Kirk et al. 2021).

The model of offering RGCS to individuals allows for cascade screening of genetic relatives, especially in the common scenario of the tested individual having no family history of AR conditions. It can also provide greater utility if the tested individuals change partners. Furthermore, it provides greater certainty for those who do have a family history or are at increased risk for conditions more common in certain population groups, such as those with Ashkenazi Jewish (AJ) ancestry (Kraft et al. 2019). Compared to the general population, individuals of AJ ancestry are at increased risk for being a genetic carrier of certain AR conditions, with one in five AJ individuals carrying a mutation for at least one of these AR conditions (Gross et al. 2008; Mitchell et al. 1996).

This disproportionate incidence and severity of AR conditions in the AJ community prompted the inception of the first targeted population genetic carrier screening programs focussing on the pre-conception period for Tay-Sachs disease (TSD), in 1971 in the USA (Kaback 2001). This model has since been successfully implemented within the Jewish community in a number of countries, including Australia, Canada, USA, and Israel, for over 30 years (Bajaj and Gross 2014; Barlow-Stewart et al. 2003; King and Klugman 2018; Klugman and Gross 2010; Mitchell et al. 1996). The range of conditions included has gradually increased over time in programs worldwide as their genetic basis was identified and advances occurred in technology, making broader genetic carrier testing possible (Vallance and Ford 2003). These RGCS programs within the Jewish community focus on the pre-conception period and those that have been most successful have been developed as community genetics programs in partnership with the local Jewish community. Their implementation in America and Canada has resulted in a 90% decline in TSD live births over a 20-year period (Barlow-Stewart et al. 2003; Kaback 2001). Health outcomes have been achieved over an extended period of time, with no babies with TSD having been born to genetic carriers identified through the programs in Australia or Israel (Broide et al. 1993; Lew et al. 2012).

Consideration of how best to reach those in the pre-conception target group led to RGCS being offered to senior high school students in both Quebec (Canada), and Sydney and Melbourne (Australia) as it allows an opportunity for education on reproductive options and family planning (King and Klugman 2018; Mitchell et al. 1996). Judicious design of the screening program can result in the efficient delivery of carrier testing. Lew et al*.* (2012) estimated that by offering a screening program in just five senior high schools operated by the Jewish community in Sydney, 50–70% of this demographic would gain access to genetic carrier screening. The RGCS program has been offered in high schools in Sydney since 1995; followed by its replication in Melbourne in 1997 (Gason et al. 2003; King and Klugman 2018). Sydney has a large Jewish population of approximately 36,000 individuals, the majority of whom have AJ ancestry (Australian Bureau of Statistics 2016; King and Klugman 2018). However, this number may be an under-representation given individuals’ reluctance to identify as Jewish due to historical persecution of this community (Graham and Waterman 2005); Eckstein, unpublished report; copies available on request from http://www.jca.org.au).

Sydney’s program was developed to allow sufficient time for education on reproductive options and family planning and facilitate informed decision-making regarding testing of carrier status. The program involves a compulsory education session for all year 11 students (aged 15–17 years) at the five schools with predominantly Jewish student enrolment, followed by voluntary carrier testing a few days later on-site. Parental consent is additionally required for students under the age of 16. Samples are collected, tested, and analysed in clinically accredited laboratories. Results are then delivered to students with appropriate counselling (King and Klugman 2018). The efficacy of the education session offered in Sydney’s RGCS program for TSD (1995–1998) and for both TSD and cystic fibrosis (CF) (1998 only) prior to testing on-site at the schools was originally established by Barlow-Stewart et al. (2003). This study found students’ knowledge and positive attitude increased, and the level of concern decreased significantly after education compared to baseline scores, enabling informed consent for testing.

The number of conditions covered by the Sydney program has continued to grow since 1998: Familial dysautonomia, Fanconi anemia, and Canavan disease were added in 2003. In 2014, the list further expanded to nine conditions, with the addition of Bloom syndrome, Niemann-Pick disease, Glycogen Storage disease 1a, and Mucolipidosis type IV (as recommended by the American College of Obstetrics and Gynaecology (American College of Obstetrics and Gynecology 2004; Monaghan et al. 2008). Given the changes in the program with these additional conditions, in 2014 we repeated the Barlow-Stewart et al. (2003) study. As in the original study, we performed measurements immediately pre-education, immediately post-education, and 12-month post-testing. Our aims in this current study were as follows: (1) assess the efficacy of the education session regarding expanded RGCS for nine conditions in terms of knowledge, attitudes, and concerns compared to that for TSD alone; (2) assess knowledge, attitudes, and to measure informed consent in regard to student testing choice when presented with an expanded testing panel; and (3) determine knowledge at baseline in 2014 compared to that in 1995 as a proxy for knowledge within the community over 20 years.

## Materials and methods

### Sample and recruitment

The study was undertaken in five senior high schools in Sydney, where the majority of attending students were known to be of AJ descent. Nearly all year 11 students (whether or not they were AJ) participated in an initial education session and were then invited to take part in this study; all these were provided with a participant information sheet and consent form. Additional written parental consent was required for participation by students under 16 years of age.

### Ethics approval

This study was subject to review by the Human Research Ethics Committee (HREC) at The University of Sydney. Ethics approval was granted prior to study commencement.

### Education

A genetic counsellor presented the education sessions, which took approximately 40 min. The core content remained the same as previous years, covering information about the conditions screened for, founder mutations, recessive inheritance, carrier frequencies, and reproductive options. The content was updated to cover the additional conditions.

### Data collection

Anonymous, coded, longitudinal, hard-copy questionnaires adapted from those used in the Barlow-Stewart et al. (2003) study were used to measure students’ knowledge, attitudes, and concerns at three time-points: immediately before the education session (T1), within 10 min after the education session (T2) and 12 months post-education and testing (T3). To ensure students’ confidentiality and anonymity, the questionnaires were de-identified and only available to the research team by individual alphanumeric codes. The school held the only link between the students’ identity and their corresponding code, allowing longitudinal data to be recorded while maintaining the students’ confidentiality.

### Measures

Knowledge was assessed through 23 questions concerning general genetics and knowledge of the inheritance patterns of conditions included in the RGCS program. Twenty of these questions included a three-point Likert scale (agree/disagree/unsure), and three included hypothetical scenarios with a four-point Likert scale specific to each question. Attitudes were assessed through four statements about genetic testing and the intended use of results. Feelings of concern about potentially being identified as a genetic carrier were assessed through twelve statements (see Supplementary File 1). Attitudes and concerns were scored using a three-point Likert scale agree/disagree/unsure. The T1 questionnaire included demographic questions (age, gender, and ancestry) and whether, or not, students were studying biology. The T2 questionnaire asked if the student was likely to agree to have testing in a few days at the school. The T3 questionnaire did not ask them about their result as there was a delay in result return for students at several schools due to external factors. All three questionnaires included an open-ended comment section at the end for feedback on the screening program and this study.

Fourteen [knowledge (*n*=10) and attitude (*n*=4)] questions were asked of both the 1995 (Barlow-Stewart et al. 2003) and the 2014 cohorts, with minor changes in wording to clarify or avoid misinterpretation, or to address inclusion of testing for the additional conditions. Four concern questions were also asked of both student cohorts with similar minor modifications to wording; however, scales differed, with a three-point Likert scale in 1995 (a lot/a little bit/not at all) but in 2014 changed to (agree/disagree/unsure).

### Data analysis

Both Jewish and non-Jewish students were included in the analyses. As per the previous study, students’ answers to the knowledge and attitude/concern questions were dichotomised. Correct knowledge answers were coded as 1, and both “incorrect” and “unsure” were coded as 0; agreement with each attitude question, or indicating worry for each concern question, was coded as 1, and classified as ‘positive’ attitude or ‘high concern’ respectively. Good knowledge, positive attitude, and high concern scores were calculated as greater than the median for the scale.

Statistical Package for the Social Sciences 22 (SPSS; IBM Corp., Armonk, NY) software was used to analyse the data, assess its distribution and apply Pearson’s chi-square, Mann-Whitney U, and Wilcoxon signed-rank tests where appropriate. Multiple linear regression modelling was used to explore associations and correlations among continuous variables. The significance level was set at *p* < 0.05, two-sided.

## Results

### Demographics

Out of a total of 332 students, 195 students completed both questionnaires at T1 and T2. Of the 195 students, 25 identified as non-Jewish; 101 were female; 43 studied biology. One hundred and sixty-five students completed and returned their questionnaire at T3 (RR = 85%). Of the students who participated at T3, 18 identified as non-Jewish; 90 were female and 36 reported study of biology (Table 1). Incomplete questionnaires were included. There was no statistically significant difference in the gender (*X*^2^=3.250, df=1, *p*=0.071) and Jewish (AJ)/non-Jewish profile (*X*^2^=3.506, df=1, *p*=0.61) of students who completed a survey at T1/T2 andT3, and those who did not.

**Table 1:** Demographics

	Pre-education (T1)/ post-education (T2) Jewish (non-Jewish)	12 months post-education (T3) Jewish (non-Jewish)
Total number	170 (25)	147 (18)
Gender		
Female Male	91 (10) 79 (15)	83 (7) 64 (11)
Age		
15 years 16 years 17 years	2 (2) 125 (20) 43 (3)	1 (2) 109 (13) 37 (3)
Study biology		
Yes No	32 (11) 138 (14)	28 (8) 119 (10)

### Reliability of scales

The scales measuring knowledge and attitudes were internally consistent (*α* = .995 for knowledge and *α* = .839 for attitude).

### Data distribution

The knowledge, concern, and attitude data collected at T1 was not normally distributed. Therefore, non-parametric statistical tests were used and students’ scores above the median at T1 were used as a threshold measure of good knowledge, concern, and positive attitude.

### Efficacy of education

Immediately after education, we observed significant increases in the number of students with good knowledge (above 12/23) (*p*=0.005) and positive attitudes (4/4) (*p*<0.001), and a concomitant decreased level of concern (<4/12) (*p*<0.001) (Table 2).

**Table 2:** Student outcomes pre- and immediate post-education

Outcome	T1	T2	Significance (*p*-value)
Median knowledge score (out of 23)	12 (IQR 8,15)	20 (IQR 17,21)	**<0.005**
Percentage with good knowledge	47%	93%	**<0.001**
Median attitude score (out of 4)	4 (IQR 3,4)	4 (IQR 4,4)	**<0.001**
Percentage with positive attitude	70%	84%	**<0.001**
Median concern score (out of 12)	4 (IQR 1,7)	1 (IQR 0,4.75)	**<0.001**
Percentage with high concern	42%	25%	**<0.001**

Values of *p*<0.05 are shown in boldface

#### Knowledge

**Fig. 1:**
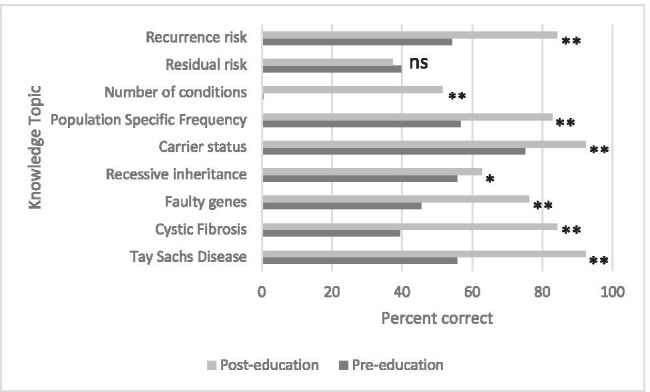
Knowledge of genetics topics over time, score by topic (% correct) for 2014 data. Significance represented by **p<0.001, *p<0.05, ns represents not significant

**Fig. 2:**
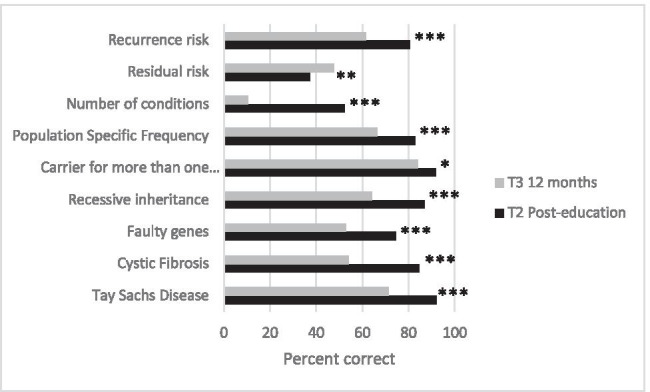
Retention of knowledge of nine genetics topics, score by topic. Percentage of Jewish and non-Jewish students (*n*=161) who answered questions in each topic area correctly. *p<0.05, **p<0.01, ***p<0.001 Wilcoxon signed-rank test for comparison of T2 (immediately post-education) and T3 (~12 months post-education) data

For eight of the nine concepts assessed, students’ knowledge increased significantly as a result of education (*p*<0.05). The one area in which students’ level of knowledge did not improve was the residual risk that testing might not identify some carriers (*p*=0.413) (Fig. 1). Although 40% of students answered the question correctly at T1, with 38% correct at T2; a significant proportion of students (42%) answered ‘unsure’ prior to education, while only 17% were unsure post-education (*p*<0.001). Therefore, a greater proportion of students answered this question incorrectly at T2 (45%) than at T1 (18%) (*p*<0.001) despite the education session (Fig. 2).

#### Attitude

Post education (T2), 138/155 (89%) of all students indicated an intention to proceed to testing and >90% had a positive attitude towards the program (Table 3). Of participants who identified as Jewish, 124/135 (92%) indicated that they would be likely to take up testing a few days later. There was no association between intention to test and good knowledge score; however, there was a trend between intention to test and positive attitude (Fisher exact test *p*=0.079).

**Table 3:** Percentage of students who answered “agree” to questions about their attitudes and concerns pre- and post-education

	T1	T2	Significance (*p* value)
Attitude			
Support genetic carrier testing in high school	83	90	**<0.005**
Would tell partner if found to be a genetic carrier	95	97	0.368
Support offering prenatal testing to carrier couples	90	96	**<0.020**
Support offering genetic carrier testing for all conditions available regardless of ancestry	83	90	**<0.010**
Concern			
If found to be a carrier for ***one*** condition, would feel:			
Worried about own health	44	21	**<0.001**
Unhealthy	19	15	0.117
Angry	16	16	1.000
Scared	48	38	**<0.010**
Depressed	15	14	0.835
If found to be a carrier for ***two*** conditions, compared to only one condition, would feel:			
More worried about own health	64	24	**<0.001**
More unhealthy	40	22	**<0.001**
More angry	24	19	0.170
More scared	49	36	**<0.001**
More depressed	22	15	**<0.010**
More unhealthy than peers	31	17	**<0.001**
More worried than peers	55	45	**<0.050**

Values of *p*<0.05 are shown in boldface

#### Concern

After education, students’ self-predicted concern about finding out they are a carrier was significantly lower (Table 3). At T1, 40.5% of students predicted they would be more concerned overall if they were found to be a carrier for two conditions rather than for one, compared to only 25.3% at T2 (*p*<0.001). Prior to education, good knowledge was associated with reduced concern (χ^2^ 4.90, (1), *p*<0.05); however, no correlation was observed at T2 (χ^2^ 0.41, (1), *p*=0.52).

### Knowledge retention

Significant retention of knowledge was measured 12 months post-education in the 161 students who completed all three questionnaires (T1, T2, and T3). Overall, 78% of the sample had retained ‘good’ knowledge 1 year after participating in the program with a median score of 15/23. While this was significantly higher than the baseline score of 12/23 (*p*<0.001) it was significantly reduced from that achieved immediate post-education: 20/23 at T2 (*p*<0.001). Importantly, there was a significant increase in the knowledge regarding residual risk over the score at T2 (Fig. 2).

#### Attitudes at 12 months post-education

With respect to the four conserved attitude questions between the 1995 and 2014 cohorts, the majority of the students (>92%) reported positive attitudes immediately after (T2), and 12 months after education and screening (T3) (>81.8%) (Table 4). Positive attitudes for all four conserved questions had significantly decreased 12 months post-education (*p*<0.01).

**Table 4:** Percentage of Jewish and non-Jewish students (*n*=143) who answered ‘agree’ to attitude questions at T2 (immediately post-education) and T3 (12-months post-education)

Core attitude question	Positive attitude (T2)	Positive attitude (T3)	Significance (*p* value)
*‘You will tell your partner when you are in a relationship’*	94.4%	83.9%	***p <0.001***
*‘Everyone should be able to have genetic carrier testing for every condition that is available even if the test is not relevant to them based on their ancestry’*	90.2%	81.8%	***p =0.019***
*‘If both partners in a couple carry the faulty gene for a condition, testing during pregnancy should be offered’*	96.5%	86.7%	***p =0.002***
*‘High school is a good time for genetic carrier testing to be offered’*	95.1%	86.0%	***p=0.012***

Values of *p*<0.05 are shown in boldface

#### Concern at 12 months post-education

Of the 138 Jewish and non-Jewish students who completed the questions about concern, median concern scores decreased between pre-education and immediately post-education (T1 median score = 4 (IQR 1,7); T2 median score 1 (IQR 0,4)). Although the median concern score increased at 12 months post-education (T3 median score 2 (IQR 1,3)), it did not increase to baseline levels, and the change between T2 to T3 median scores was not significant (*p*=0.214). Indeed, only 22% of students exhibited high concern scores at T3.

### Measure of informed consent

In a previous study by Marteau and colleagues, individuals were considered to have made an informed choice if they had good knowledge, a positive attitude and chose testing, or they had good knowledge, a negative attitude and chose not to test (Marteau et al. 2001). Students were not asked about their test result in T3 due to delays in result reporting during the study period. Therefore, in this study, we used the individual’s statement of their likelihood to have testing as a proxy for choosing to have testing; individuals who indicated they were not likely to have testing or who were unsure were used as a proxy for not having testing. Although the intention to test is hypothetical, we believe it represents a willingness to act based on the content/evidence presented at the education session. At T2, of the 138/155 (89%) students who indicated that they were likely to have testing, 114/138 (83%) had good knowledge and a positive attitude. Of those who indicated they were unsure or likely not to have testing 6/17 had good knowledge and a negative attitude. Therefore, 120/155 (77.4%) were likely to make an informed choice regarding their decision to test or not test.

### Influencing factors on student outcomes

No significant relationship was found between identifying as Jewish and any of the outcomes of knowledge, attitude, and concern before or after education or 12 months later. However, there was an association between being male and having a less positive attitude pre- and post-education and a lower concern at T1. For those studying biology there was an association with having a more positive attitude at T2, with a trend towards increased knowledge at T2 and retention of knowledge at T3 (see Table 5).

**Table 5:** Factors influencing student outcomes

Association	Gender	Jewish	Studying biology
Timepoint 1			
Good knowledge score (*n*=184)	*χ*^2^ 0.48 (1) p = 0.49	*χ*^2^ 1.06 (1) *p* = 0.30	*χ*^2^ 1.65 (1) *p* = 0.20
Positive attitude score (*n*=194)	***χ***^**2**^**5.13 (1)** ***p*** < 0.05*	*χ*^2^ 1.01 (1) *p* = 0.32	*χ*^2^ 0.92 (1) *p* = 0.34
High concern score (*n*=189)	***χ***^**2**^ **7.11(1)** ***p*** < 0.010*	*χ*^2^ 1.39 (1) *p* = 0.24	*χ*^2^ 0.70 (1) *p* = 0.40
Timepoint 2			
Good knowledge score (*n*=195)	*χ*^2^ 0.99 (1) *p* = 0.32	Fisher *p* = 0.22	Fisher *p* = 0.076
Positive attitude score (*n*=194)	***χ***^**2**^**7.14 (1)** *p*< 0.01*	Fisher *p* = 0.31	***χ***^**2**^ **4.59 (1)** ***p*** < 0.05*
High concern score (*n*=181)	*χ*^2^ 1.09 (1) *p* = 0.30	*χ*^2^ 1.00 (1) *p* = 0.32	*χ*^2^ 3.41 (1) *p* = 0.07
Timepoint 3			
Good knowledge score (*n*=161)	*χ*^2^ 1.25 (1) *p* = 0.26	*χ*^2^ 2.92 (1) *p* = 0.59	*χ*^2^ 3.75 (1) *p* = 0.053
Positive attitude score (*n*=153)	*χ*^2^ 0.19 (1) *p* = 0.67	*χ*^2^ 0.23 (1) *p* = 0.63	*χ*^2^ 0.13 (1) *p* = 0.72
High concern score (*n*=156)	*χ*^2^ 0.83 (1) *p* = 0.77	*χ*^2^ 0.13 (1) *p* =0.91	*χ*^2^ 1.49 (1) *p* = 0.22

Fisher represents a Fisher exact test; * p<0.05. Values of p<0.05 are shown in boldface

### Comparison between 1995 and 2014 student cohorts at baseline

A total of 321 year 11 students from the four schools submitted pre- and post-education questionnaires in 1995 of which 279 were complete (Barlow-Stewart et al. 2003). Median baseline knowledge about TSD and genetic concepts has increased significantly between 1995 and 2014 (z=11.15, *p*<0.001), although still not to the level achieved after education. Baseline attitude also improved over the 19 years from a median score of 3 (IQR 2,3) in 1995 to 3 (IQR 3,3) in 2014 out of a possible score of 3 (*p*=0.024) (Table 6).

**Table 6:** Students’ median scores for outcomes in 1995 compared to 2014

Outcome	1995^a^ median (IQR)	2014^b^ median (IQR)	Significance (*p* value)
Pre-education (T1)			
Knowledge score (out of 10)	3 (2,4)	6 (4,7)	**<0.001**
Attitude score (out of 3)	3 (2,3)	3 (3,3)	**0.024**
Post-education (T2)			
Knowledge score (out of 10)	9 (8,9)	10 (8,10)	**<0.001**
Attitude score (out of 3)	3 (3,3)	3 (3,3)	**0.122**

^a^*n*=279 knowledge, *n*=282 attitudes in 1995, ^*b*^*n=*195 knowledge, *n*=186 attitudes in 2014. *IQR* interquartile range. Values of *p*<0.05 are shown in boldface

**Fig. 3:**
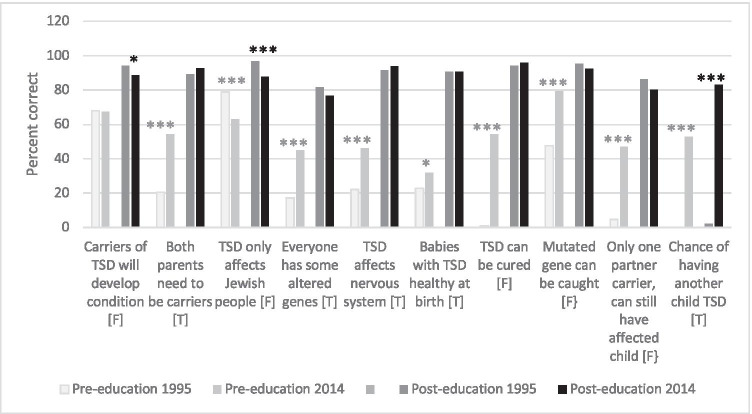
Ten knowledge questions conserved between 1995 and 2014. (F) = false, (T) = true. *p<0.05, ***p<0.001 for comparison of the percentage of students who correctly answered each knowledge question in 1995 vs. 2014 using *χ*^2^ test. Grey and black asterisks represent a significant difference between pre-education and post-education results respectively

Students in 1995 had a better baseline understanding of one of the ten conserved knowledge questions compared to 2014 (Fig. 3). This question was ‘only individuals of Jewish descent can have a child with TSD’ (false) (*χ*^2^ 4.62, (1), *p*<0.001). However, a larger proportion of students answered eight of the remaining nine questions correctly in 2014. Of these, six are specifically related to knowledge of TSD.

### Impact of expansion from one to nine conditions

Overall, median knowledge after education (at T2) was significantly higher when testing was offered for nine conditions rather than TSD alone (Table 6). For individual questions, a significantly larger percentage of students correctly answered one of the ten conserved knowledge questions related to TSD in 2014 compared to the 1995 cohort (*χ*^2^ 328, (1), *p*<0.001): ‘If a couple already have a child with TSD, there is the same chance they will have another child with the condition (T)’. In comparison, a significantly larger proportion of students from 1995 answered the following questions correctly, as compared to students in 2014: (i) Only Ashkenazi Jewish people can have a baby with TSD or other conditions common in the Jewish community (false) (*χ*^2^ 14.6, (1), *p*<0.001), (ii) A person who is a carrier of the faulty gene which causes TSD will develop TSD at some time in their life (false) (*χ*^2^ 4.79, (1), *p*=0.030) (Fig. 3). For both 1995 and 2014 cohorts, post-education results indicated that over 75% of students answered all nine questions correctly, except for the 1995 cohort where students did not understand the chance of having another child with TSD would be the same for each child if the parents were both carriers.

## Discussion

Concerns have been raised about the impact of including an ever-expanding number of conditions in RGCS on achieving informed consent (Henneman et al. 2016). Indeed, Ioannou et al. (2010) compared the effects of a seven-disease screening panel with a single-disease panel (TSD) in Jewish high schools in Melbourne (Ioannou et al. 2010). They found that increasing the number of conditions resulted in decreased knowledge and increased predicted negative feelings if found to be a carrier of one of the conditions. The Melbourne findings raised relevant concerns regarding the capacity of the Sydney Jewish high school program to ensure the students are providing informed consent when offered RGCS for a similarly expanding screening panel of nine conditions, which was one of the reasons we performed this present study. The results presented here show that we have successfully provided education about an increased number of conditions without compromising the acquisition of knowledge, and without increasing students’ prediction of negative feelings regarding being a carrier.

The majority of the high school students who participated in this study were able to provide informed consent to screening for the expanded panel of nine conditions, based on that measured by levels of knowledge, attitudes, and intention to test (Marteau et al. 2001). These findings confirmed that through an education-driven program, the high school setting is associated with higher test uptake, addressing concerns raised by the Melbourne study (Ioannou et al. 2010) as well as the capacity of adolescents to give informed consent (Frumkin and Zlotogora 2008; Harel et al. 2003; Marteau et al. 1992). Education is a key to this success. The RGCS high school program such as that offered in Sydney is underpinned by education that equips individuals to make informed choices about test uptake and reproductive options (Kaback et al. 1993; Kaback 2001; King and Klugman 2018). Inadequate education has been associated with increased anxiety, reduced recall of genetic concepts, and reduced understanding of the implications of genetic testing (Gason et al. 2003; Hegwer et al. 2006).

Establishing adequate short-term knowledge levels and positive attitudes in participants is important. However, as the information delivered is likely to be more relevant in subsequent years, the retention of knowledge over time and the long-term impact of the program on students’ attitudes are particularly significant. Our study has shown that good levels of knowledge were retained 12 months later, even when nine conditions were included. As the number of conditions tested increases, the number of individuals identified as carriers also increases (Fares et al. 2008). This indicates that in increasing the number of conditions tested from five to nine, Sydney’s program is likely to identify more carriers, and therefore the education session will be relevant to a larger proportion of students than in previous years. The one area in which participants’ knowledge did not increase significantly was in understanding that testing might not identify some carriers, leading to a residual risk. Understanding of this concept of residual risk was poor and it was the only area in which less than 50% of participants answered correctly. Poor understanding of residual risk may be the product of increasing media attention and exposure to false depictions of DNA testing in film and television (Hylind et al. 2018). The importance of highlighting residual risk with advancing technology has been expressed previously (Mitchell et al. 1996). More focus on residual risk has since been made in light of these findings, to further increase student understanding of this topic and to increase the efficacy of the education session.

Comparison of post-education data from 2014 with student outcomes from 1995 clearly demonstrated that, despite the increased number of conditions covered, median knowledge and attitude scores after education were significantly higher than for just a single genetic condition (TSD). Furthermore, a significant increase in knowledge was seen between the cohorts for a conserved question regarding the risk of being a genetic carrier in specific populations. Population-specific risk is a particularly important component of the education session, to ensure informed consent and understanding of the relevancy of testing to this population. Awareness of why screening is relevant to the AJ community is an important measure of the success of the program (King and Klugman 2018).

### Baseline student knowledge as a proxy for community knowledge over 20 years

Measurement of baseline knowledge of the students after almost 20 years of annual education is a proxy for community knowledge. Knowledge of TSD and genetic concepts were higher in 2014 than in 1995 before they received this dedicated education, despite the fact that no babies have been born with TSD in this community in that period (Lew et al. 2012). Attitudes towards testing and the use of results also improved over time.

However, when the program first began, a greater proportion of students understood that non-Jewish couples can have a child with TSD, than after 20 years of education. This is concerning as it suggests that the provision of testing specifically to the Jewish population may have promoted the false belief within the community that TSD only occurs in the Jewish population. This is despite the opposite message being imparted through the annual education session. The finding that this misunderstanding is rectified after education supports this supposition. While baseline knowledge and attitudes of the community have improved significantly over time, it is evident that the education sessions are effective and significantly improve students’ outcomes to enable an informed choice. The findings of this study validate the continued implementation of education as a key component of the genetic carrier screening program.

### Retention of knowledge

The education session was effective in facilitating significantly increased understanding and retention of knowledge over time. This is particularly important as the students participating in the program are aged between 15 and 17 years, and the average age of first-time parents in Australia is 28.4 years (Hilder et al. 2014). Therefore, retention of the knowledge delivered through education is essential for appropriate use of results and understanding of reproductive ramifications. The increase and retention of knowledge post-education are indicative of effective education and communication of information to this cohort.

### Retention of attitudinal outcomes

Although the percentage of students who answered positively to attitude questions was reduced significantly 1 year following initial surveying, the percentage of students with a positive attitude remained high (>81.8%). The majority of students reported positive attitudes and positive intended use of results after education. The median attitudes remained unchanged over the course of the study, signifying retention of positive attitudes over time.

### Implications for practice

Integration of genomic technologies into the high school genetic screening program is expected to facilitate expanding testing to even more AR conditions, pending technical and clinical evaluations for effectiveness, utility, and acceptance. Thus, the findings from this study will become increasingly relevant with even more expanded RGCS being offered to high school students. The number of individuals identified as carriers is likely to increase and demand for genetic counselling and education is likely to become more relevant to more students. It is estimated that offering a panel of sixteen conditions will result in 30% of the cohort being identified as a carrier for at least one of these conditions (Scott et al. 2010). Therefore, establishing that retention of education is feasible in the context of multi-disease carrier screening programs has extensive practice implications.

### Limitations

Upon completion of data collection, a small number of students were identified as not having received their genetic carrier screening results from the laboratory (<10). Due to the de-identified nature of the data collection, these students could not be excluded from the analysis. However, these individuals still received education and participated in testing, but it is possible that a lack of knowledge of carrier status could have an impact on student outcomes. A further limitation for this research is that the genetic carrier screening program was, at the time, offered in only five high schools in Sydney with predominantly Jewish student enrolments. Even though this cohort represents more than an estimated half of all Jewish students in this community population, it cannot be concluded that our findings are automatically representative of Jewish students attending other schools. In addition, differences in access to information and education between 1995 and 2014, since the advent of the internet, might be a limitation in comparing knowledge of students before and after the education session.

Finally, by comparing data from 20 years ago, findings may not solely reflect the impact of expanded screening. Other changes may have influenced findings, such as a change in the genetic counsellor delivering the education, as well as access to further information from the Internet and media. An additional source of information might have been the students’ parents, some of whom themselves might have been participants in the original program of 20 years earlier, and this might also potentially influence the students’ stated intentions.

### Future research

As the Sydney Jewish high school screening program evolves to accommodate developing genomic technology and relevant changes to the education session are made, continual evaluation of the efficacy of the education is important to ensure the program’s integrity is maintained. Furthermore, research examining concern levels in students before and after education is necessary to supplement data missing in this study.

The findings that expanded screening did not result in decreased knowledge or attitude scores post-education, are in conflict with those found by Ioannou et al. (2010) and may require further investigation to discover the following: (i) whether there are any identifiable elements of Sydney’s education session that differ from other programs, and which may have contributed to its success, and (ii) how these elements could be extrapolated for inclusion in other, broader educational applications to other communities.

In 1999–2000, we undertook a preliminary evaluation of schools-based carrier screening for cystic fibrosis (CF), a condition common in a particular rural community. This community had few (or even no) AJ members, but the education nevertheless covered three conditions relevant to the Australian community at large (viz. CF, TSD, and thalassaemia). As for the Jewish schools, evaluation utilised the original survey of Barlow-Stewart et al. (2003) at T1, T2, and T3, but modified for CF only. We found essentially the same results (data not shown) as described in the original Barlow-Stewart et al. (2003) paper, suggesting that our methodology is robust and that our approach is not restricted just to metropolitan urban AJ communities, or just to TSD, and could be modified to ensure relevance to the target community. This is an avenue for further research as we have not yet assessed it in communities where, for example, there are high rates of consanguinity.

A long-term follow-up study of the sample may be useful to examine the retention of knowledge and educational outcomes, when participants of the program begin to make reproductive decisions. Such a study may also identify the clinical utility of the screening provided, as well as establish the use of results in family planning.

Finally, there is limited literature published regarding the impact of the Jewish high school screening program on the incidence of conditions other than TSD. Epidemiological studies relevant to the AR conditions included in the program may be beneficial in determining the success of the program in reducing disease incidence.

## Conclusion

The provision of annual education via high schools to the Jewish community for two decades has significantly improved the overall baseline knowledge (Barlow-Stewart et al. 2003). Expansion of the Sydney Jewish high school genetic carrier screening program to nine conditions did not negatively impact student outcomes. Effective education delivery was provided for nine conditions to enable informed consent. Comparison of student outcomes to research done when the program offered screening for only one condition revealed no significant difference in knowledge and understanding of the education delivered despite the expansion in the testing panel. The education resulted in significant retention of knowledge and positive attitude 12 months post-education and screening. These findings may be used to inform similar targeted population screening programs based on this high school screening model to inform the implementation of other population genetic carrier screening programs.

## Supplementary Information

Supplementary File 1: Surveys T1 and T2 and T3 (PDF 1.53 mb)
