# Supplementary material for: Evaluating the model of offering expanded genetic carrier screening to high school students within the Sydney Jewish community
Source: J Community Genet. 2021 Nov 30;13(1):121–31. doi: 10.1007/s12687-021-00567-8 (PMC8799788; doi:10.1007/s12687-021-00567-8)
Supplement: Supplementary file 1 — Surveys T1 and T2 and T3 (PDF 1.53 mb) [file 12687_2021_567_MOESM1_ESM.pdf]

THE EFFECTIVENESS OF EDUCATION IN THE GENETIC CARRIER TESTING PROGRAM

RESEARCH

SURVEY 1 2014

CODE NO. __________

We would appreciate it if you would complete this survey for our research into the effectiveness of education in the new genetic carrier testing program. The education session today has been recently updated. We are studying whether the information provided in the education session helps students make informed decisions about whether or not to have genetic carrier testing offered at school in a few days time.

This information is important to further development of future education sessions and carrier testing programs.

Being in this study is completely voluntary. You are under no obligation to fill out this survey. Being in this study is separate to having the genetic testing itself, and if you choose not to participate you will still have full access to all aspects of the usual screening program.

The survey should only take about 10 minutes to complete. Please answer all the questions as they apply to you. This survey is not testing you, but is assessing the education session. Just select whichever answer you think is best for each question. Please hand it in to your teacher or the educator when you have completed it.

All of your answers are completely anonymous and confidential. The researchers will at no time be able to identify which questionnaire belongs to which student.

We appreciate the time and support you are giving to this important research.

PROF. KRISTINE BARLOW-STEWART
Director, Master of Genetic Counselling Program
Sydney Medical School – Northern
Royal North Shore Hospital
The University of Sydney

KAYLEY BROOKER
Master of Genetic Counselling Student,
The University of Sydney

DR YEMIMA BERMAN
Clinical Geneticist/ Associate Lecturer,
Royal North Shore Hospital
The University of Sydney

PROF. LESLIE BURNETT
Clinical Pathologist/ Clinical Professor,
PaLMS, Royal North Shore Hospital
The University of Sydney

In partnership with
The Sydney Jewish Community, PaLMS, NSW Health Pathology, and RNSH Clinical Genetics

INSTRUCTIONS: Colour the circle for only one number for each statement or question and do not skip any items. Choose the answer you think is best. If you change your mind, cross out your first mark carefully.

SOME GENERAL QUESTIONS THAT ARE IMPORTANT FOR THE ANAYLSIS OF THE SURVEY (Questions a - e)

(a) Gender Male ① Female ② **(b) Age** 15 yrs ① 16 yrs ② 17 yrs ③

(c) Are you Jewish? Yes ① No ② **(d) Are you studying biology as a HSC subject?** Yes ① No ②

(e) Australia is a multicultural society made up of people from many parts of the world. Please indicate if any of your parents, grandparents or great grandparents are from any of the following ancestry (you may need to colour more than one circle as your ancestors may have come from different parts of the world):

① Northern European (including England, Scotland, Wales, and Ireland) ② Middle Eastern

③ Asian (including China, Hong Kong, Japan and S.E. Asian Countries ④ Indian

⑤ Southern European (such as Greece and Southern Italy) ⑥ Other (specify) ____________________

KNOWLEDGE OF TAY-SACHS DISEASE AND OTHER GENETIC CONDITIONS THAT OCCUR FREQUENTLY IN THE AUSTRALIAN COMMUNITY	AGREE	DISAGREE	UNSURE
1. Tay-Sachs disease is a disorder that affects the nervous system	①	②	③
2. Babies with Tay-Sachs disease are healthy at birth	①	②	③
3. Cystic Fibrosis is a disorder that affects a person's lungs and digestive system	①	②	③
4. Tay-Sachs disease can be cured	①	②	③
5. Tay-Sachs disease can be 'caught' from others	①	②	③
6. Everyone is born carrying a number of faulty genes in the cells of their body	①	②	③
7. Some faulty genes are more common in people with particular ancestry	①	②	③
8. For historical reasons, cultural practices or geographic barriers, certain genetic conditions are more common in some communities in Australia than in the general population	①	②	③
9. A person who is a carrier of the faulty gene which causes Tay-Sachs disease will develop Tay-Sachs at some time in their life	①	②	③
10. A person who is a carrier of the faulty gene which causes Cystic Fibrosis will develop Cystic Fibrosis at some time in their life	①	②	③
11. If both partners in a couple have been tested and only one partner carries faulty gene for Tay-Sachs disease, they can still have a baby with Tay-Sachs disease	①	②	③
12. Conditions like Tay-Sachs disease, Cystic Fibrosis, Gaucher disease and Familal Dysautonomia are all caused by the child inheriting a particular faulty gene from BOTH their mother and their father	①	②	③
13. A person can be a carrier of the faulty genes for more than one genetic condition	①	②	③
14. If you are of European ancestry, and Jewish (Ashkenazi Jewish), you are at high risk (1 in 25 chance) for carrying the faulty gene for Tay-Sachs disease	①	②	③
15. Only Ashkenazi Jewish people can have a baby with Tay-Sachs disease or other conditions common in the Jewish community	①	②	③
16. If a person is of European or UK ancestry (but not Jewish), there is a high risk (1 in 25 chance) that they are a carrier for the faulty gene for Cystic	①	②	③

Fibrosis

17. If a person is of Sth European, Asian, Indian or Middle Eastern ancestry, there is a high risk (1 in 25 chance) that they are a carrier for the faulty gene for Thalassaemia	✏	✂	✂
18. The Jewish school genetic carrier testing program screens for 5 different genetic conditions	✏	✂	✂
19. The genetic carrier testing is completely accurate and there is no degree of uncertainty in the test result	✏	✂	✂
20. If the test shows that you are not a carrier for the faulty gene for a particular condition you cannot have a child with that condition	✏	✂	✂

FOR EACH QUESTION BELOW, JUST TICK THE BOX YOU THINK IS RIGHT

21. For a couple to have a child with Tay-Sachs Disease, who needs to be a carrier of Tay-Sachs Disease?
- One partner only
- Neither partner
- Both partners
- Unsure

22. If a couple already have a child with Tay-Sachs Disease, what is their chance of having another child with Tay-Sachs Disease?
- The same with each child born
- Less with each child born
- More with each child born
- Unsure

23. What is the chance of being a carrier of the faulty gene for genetic conditions like Tay-Sachs Disease if you are Ashkenazi Jewish, compared with the general community?
- The same
- Higher
- Lower
- Unsure

24. Unlike the tests being offered through the school testing program, other genetic tests are available that may have implications for your own health. How important are these tests for *you*?
- Not at all
- Somewhat
- Moderately
- Very

**ATTITUDES TOWARDS CARRIER GENETIC TESTING**	**AGREE**	**DISAGREE**	**UNSURE**
25. If you found out that you were a carrier for a faulty gene that may cause a particular condition in your children:			
a. You would tell your partner if you were in a relationship	✏	✂	✂
b. You think that you will feel unhealthy	✏	✂	✂
c. You think that you will feel worried about your own health	✏	✂	✂
d. You think that you will feel angry	✏	✂	✂

e. You think that you will feel scared	✎	✂	✁
f. You think that you will feel depressed	✎	✂	✁

26. If you found out that you were a carrier of two different faulty genes that may cause two different conditions in your children:

a. You would tell your partner if you were in a relationship	✎	✂	✁
b. You think that you will feel more unhealthy than if you carried one faulty gene	✎	✂	✁
c. You think that you will feel more worried than if you carried one faulty gene	✎	✂	✁
d. You think that you will feel more angry than if you carried one faulty gene	✎	✂	✁
e. You think that you will feel more scared than if you carried one faulty gene	✎	✂	✁
f. You think that you will feel more depressed than if you carried one faulty gene	✎	✂	✁

27. If you found out that you were a carrier of faulty genes that may cause two different conditions in your children:

a. You think that you will feel more unhealthy than your peers	✎	✂	✁
b. You think that you will feel more worried than your peers	✎	✂	✁

CHOICES ABOUT GENETIC TESTING	AGREE	DISAGREE	UNSURE
28. Everyone should be able to have genetic carrier testing for every condition that is available even if the test is not relevant to them based on their ancestry	✎	✂	✁
29. High school is a good time to offer genetic carrier testing	✎	✂	✁
30. If both partners in a couple carry the faulty gene for the same condition, testing during pregnancy should be offered	✎	✂	✁

31. How do *you* feel about new discoveries in genetics?

Please select the *most appropriate* response for *you* to these 6 statements by circling the number for each scale:

Bored	1	2	3	4	5	6	7	Excited
Valuable	1	2	3	4	5	6	7	Worthless
Uninterested	1	2	3	4	5	6	7	Interested
Indifferent	1	2	3	4	5	6	7	Passionate
Important	1	2	3	4	5	6	7	Unimportant

32. Do you have any comments you would like to make?

____

____"

____"

YOU HAVE NOW COMPLETED THE SURVEY. THANK YOU FOR PARTICIPATING IN THIS RESEARCH.

THE EFFECTIVENESS OF EDUCATION IN THE GENETIC CARRIER TESTING PROGRAM

RESEARCH

SURVEY 2 2014

CODE NO. __________

We would appreciate it if you would complete this survey for our research into the effectiveness of education in the new genetic carrier testing program. The education session today has been recently updated. We are studying whether the information provided in the education session helps students make informed decisions about whether or not to have genetic carrier testing offered at school in a few days time.

This information is important to further development of future education sessions and carrier testing programs.

Being in this study is completely voluntary. You are under no obligation to fill out this survey. Being in this study is separate to having the genetic testing itself, and if you choose not to participate you will still have full access to all aspects of the usual screening program.

The survey should only take about 10 minutes to complete. Please answer all the questions as they apply to you. This survey is not testing you, but is assessing the education session. Just select whichever answer you think is best for each question. Please hand it in to your teacher or the educator when you have completed it.

All of your answers are completely anonymous and confidential. The researchers will at no time be able to identify which questionnaire belongs to which student.

We appreciate the time and support you are giving to this important research.

PROF. KRISTINE BARLOW-STEWART
Director, Master of Genetic Counselling Program
Sydney Medical School – Northern
Royal North Shore Hospital
The University of Sydney

KAYLEY BROOKER
Master of Genetic Counselling Student,
The University of Sydney

DR YEMIMA BERMAN
Clinical Geneticist/ Associate Lecturer,
Royal North Shore Hospital
The University of Sydney

PROF. LESLIE BURNETT
Clinical Pathologist/ Clinical Professor,
PaLMS, Royal North Shore Hospital
The University of Sydney

In partnership with
The Sydney Jewish Community, PaLMS, NSW Health Pathology, and RNSH Clinical Genetics

INSTRUCTIONS: Colour the circle for only one number for each statement or question and do not skip any items. Choose the answer you think is best. If you change you mind, cross out your first mark carefully.

KNOWLEDGE OF TAY-SACHS DISEASE AND OTHER GENETIC CONDITIONS THAT OCCUR FREQUENTLY IN THE AUSTRALIAN COMMUNITY	AGREE	DISAGREE	UNSURE
1. Tay-Sachs disease is a disorder that affects the nervous system	①	②	③
2. Babies with Tay-Sachs disease are healthy at birth	①	②	③
3. Cystic Fibrosis is a disorder that affects a person's lungs and digestive system	①	②	③
4. Tay-Sachs disease can be cured	①	②	③
5. Tay-Sachs disease can be 'caught' from others	①	②	③
6. Everyone is born carrying a number of faulty genes in the cells of their body	①	②	③
7. Some faulty genes are more common in people with particular ancestry	①	②	③
8. For historical reasons, cultural practices or geographic barriers, certain genetic conditions are more common in some communities in Australia than in the general population	①	②	③
9. A person who is a carrier of the faulty gene which causes Tay-Sachs disease will develop Tay-Sachs at some time in their life	①	②	③
10. A person who is a carrier of the faulty gene which causes Cystic Fibrosis will develop Cystic Fibrosis at some time in their life	①	②	③
11. If both partners in a couple have been tested and only one partner carries faulty gene for Tay-Sachs disease, they can still have a baby with Tay-Sachs disease	①	②	③
12. Conditions like Tay-Sachs disease, Cystic Fibrosis, Gaucher disease and Familal Dysautonomia are all caused by the child inheriting a particular faulty gene from BOTH their mother and their father	①	②	③
13. A person can be a carrier of the faulty genes for more than one genetic condition	①	②	③
14. If you are of European ancestry, and Jewish (Ashkenazi Jewish), you are at high risk (1 in 25 chance) for carrying the faulty gene for Tay-Sachs disease	①	②	③
15. Only Ashkenazi Jewish people can have a baby with Tay-Sachs disease or other conditions common in the Jewish community	①	②	③
16. If a person is of European or UK ancestry (but not Jewish), there is a high risk (1 in 25 chance) that they are a carrier for the faulty gene for Cystic Fibrosis	①	②	③
17. If a person is of Sth European, Asian, Indian or Middle Eastern ancestry, there is a high risk (1 in 25 chance) that they are a carrier for the faulty gene for Thalassaemia	①	②	③
18. The Jewish school genetic carrier testing program screens for 5 different genetic conditions	①	②	③
19. The genetic carrier testing is completely accurate and there is no degree of uncertainty in the test result	①	②	③
20. If the test shows that you are not a carrier for the faulty gene for a particular condition you cannot have a child with that condition	①	②	③

FOR EACH QUESTION BELOW, JUST TICK THE BOX YOU THINK IS RIGHT

21. For a couple to have a child with Tay-Sachs Disease, who needs to be a carrier of Tay-Sachs Disease?
    - One partner only
    - Neither partner
    - Both partners
    - Unsure

22. If a couple already have a child with Tay-Sachs Disease, what is their chance of having another child with Tay-Sachs Disease?
    - The same with each child born
    - Less with each child born
    - More with each child born
    - Unsure

23. What is the chance of being a carrier of the faulty gene for genetic conditions like Tay-Sachs Disease if you are Ashkenazi Jewish, compared with the general community?
    - The same
    - Higher
    - Lower
    - Unsure

24. Unlike the tests being offered through the school testing program, other genetic tests are available that may have implications for your own health. How important are these tests for *you*?
    - Not at all
    - Somewhat
    - Moderately
    - Very

ATTITUDES TOWARDS CARRIER GENETIC TESTING	AGREE	DISAGREE	UNSURE

25. If you found out that you were a carrier for a faulty gene that may cause a particular condition in your children:

	AGREE	DISAGREE	UNSURE
a. You would tell your partner if you were in a relationship			
b. You think that you will feel unhealthy			
c. You think that you will feel worried about your own health			
d. You think that you will feel angry			
e. You think that you will feel scared			
f. You think that you will feel depressed			

26. If you found out that you were a carrier of two different faulty genes that may cause two different conditions in your children:

	AGREE	DISAGREE	UNSURE
a. You would tell your partner if you were in a relationship			
b. You think that you will feel more unhealthy than if you carried one faulty gene			
c. You think that you will feel more worried about your own health than if you carried one faulty gene			
d. You think that you will feel more angry than if you carried one faulty gene			
e. You think that you will feel more scared than if you carried one faulty gene			

e. You think that you will feel scared

f. You think that you will feel depressed

26. If you found out that you were a carrier of two different faulty genes that may cause two different conditions in your children:

a. You would tell your partner if you were in a relationship

b. You think that you will feel more unhealthy than if you carried one faulty gene

c. You think that you will feel more worried than if you carried one faulty gene

d. You think that you will feel more angry than if you carried one faulty gene

e. You think that you will feel more scared than if you carried one faulty gene

f. You think that you will feel more depressed than if you carried one faulty gene

27. If you found out that you were a carrier of faulty genes that may cause two different conditions in your children:

a. You think that you will feel more unhealthy than your peers

b. You think that you will feel more worried than your peers

**CHOICES ABOUT GENETIC TESTING**	**AGREE**	**DISAGREE**	**UNSURE**
28. Everyone should be able to have genetic carrier testing for every condition that is available even if the test is not relevant to them based on their ancestry			
29. High school is a good time to offer genetic carrier testing			
30. If both partners in a couple carry the faulty gene for the same condition, testing during pregnancy should be offered			

31. How do *you* feel about new discoveries in genetics?

Please select the *most appropriate* response for *you* to these 6 statements by circling the number for each scale:

Bored	1	2	3	4	5	6	7	Excited
Valuable	1	2	3	4	5	6	7	Worthless
Uninterested	1	2	3	4	5	6	7	Interested
Indifferent	1	2	3	4	5	6	7	Passionate
Important	1	2	3	4	5	6	7	Unimportant

32. Do you have any comments you would like to make?

____

____"

____"

YOU HAVE NOW COMPLETED THE SURVEY. THANK YOU FOR PARTICIPATING IN THIS RESEARCH.

THE EFFECTIVENESS OF EDUCATION IN THE GENETIC CARRIER TESTING PROGRAM

RESEARCH

SURVEY 3 2014

CODE NO. __________

We would appreciate it if you would complete this survey for our research into the effectiveness of education in the new genetic carrier testing program. The education session you took part in has been recently updated. We are studying whether the information provided in the education session helps students make informed decisions about whether or not to have genetic carrier testing offered at school in a few days time.

This information is important to further development of future education sessions and carrier testing programs.

Being in this study is completely voluntary. You are under no obligation to fill out this survey. Being in this study is separate to having the genetic testing itself, and if you choose not to participate you will still have full access to all aspects of the usual screening program.

The survey should only take about 10 minutes to complete. Please answer all the questions as they apply to you. This survey is not testing you, but is assessing the education session. Just select whichever answer you think is best for each question. Please hand it in to your teacher or the educator when you have completed it.

All of your answers are completely anonymous and confidential. The researchers will at no time be able to identify which questionnaire belongs to which student.

We appreciate the time and support you are giving to this important research.

PROF. KRISTINE BARLOW-STEWART
Director, Master of Genetic Counselling Program
Sydney Medical School – Northern
Royal North Shore Hospital
The University of Sydney

KAYLEY BROOKER
Master of Genetic Counselling Student,
The University of Sydney

DR YEMIMA BERMAN
Clinical Geneticist/ Associate Lecturer,
Royal North Shore Hospital
The University of Sydney

PROF. LESLIE BURNETT
Clinical Pathologist/ Clinical Professor,
PaLMS, Royal North Shore Hospital
The University of Sydney

In partnership with
The Sydney Jewish Community, PaLMS, NSW Health Pathology, and RNSH Clinical Genetics

INSTRUCTIONS: Colour the circle for only one number for each statement or question and do not skip any items. Choose the answer you think is best. If you change you mind, cross out your first mark carefully.

KNOWLEDGE OF TAY-SACHS DISEASE AND OTHER GENETIC CONDITIONS THAT OCCUR FREQUENTLY IN THE AUSTRALIAN COMMUNITY	AGREE	DISAGREE	UNSURE
1. Tay-Sachs disease is a disorder that affects the nervous system	①	②	③
2. Babies with Tay-Sachs disease are healthy at birth	①	②	③
3. Cystic Fibrosis is a disorder that affects a person's lungs and digestive system	①	②	③
4. Tay-Sachs disease can be cured	①	②	③
5. Tay-Sachs disease can be 'caught' from others	①	②	③
6. Everyone is born carrying a number of faulty genes in the cells of their body	①	②	③
7. Some faulty genes are more common in people with particular ancestry	①	②	③
8. For historical reasons, cultural practices or geographic barriers, certain genetic conditions are more common in some communities in Australia than in the general population	①	②	③
9. A person who is a carrier of the faulty gene which causes Tay-Sachs disease will develop Tay-Sachs at some time in their life	①	②	③
10. A person who is a carrier of the faulty gene which causes Cystic Fibrosis will develop Cystic Fibrosis at some time in their life	①	②	③
11. If both partners in a couple have been tested and only one partner carries faulty gene for Tay-Sachs disease, they can still have a baby with Tay-Sachs disease	①	②	③
12. Conditions like Tay-Sachs disease, Cystic Fibrosis, Gaucher disease and Familal Dysautonomia are all caused by the child inheriting a particular faulty gene from BOTH their mother and their father	①	②	③
13. A person can be a carrier of the faulty genes for more than one genetic condition	①	②	③
14. If you are of European ancestry, and Jewish (Ashkenazi Jewish), you are at high risk (1 in 25 chance) for carrying the faulty gene for Tay-Sachs disease	①	②	③
15. Only Ashkenazi Jewish people can have a baby with Tay-Sachs disease or other conditions common in the Jewish community	①	②	③
16. If a person is of European or UK ancestry (but not Jewish), there is a high risk (1 in 25 chance) that they are a carrier for the faulty gene for Cystic Fibrosis	①	②	③
17. If a person is of Sth European, Asian, Indian or Middle Eastern ancestry, there is a high risk (1 in 25 chance) that they are a carrier for the faulty gene for Thalassaemia	①	②	③
18. The Jewish school genetic carrier testing program screens for 5 different genetic conditions	①	②	③
19. The genetic carrier testing is completely accurate and there is no degree of uncertainty in the test result	①	②	③
20. If the test shows that you are not a carrier for the faulty gene for a particular condition you cannot have a child with that condition	①	②	③

FOR EACH QUESTION BELOW, JUST TICK THE BOX YOU THINK IS RIGHT

21. For a couple to have a child with Tay-Sachs Disease, who needs to be a carrier of Tay-Sachs Disease?
    - ☐ One partner only
    - ☐ Neither partner
    - ☐ Both partners
    - ☐ Unsure
22. If a couple already have a child with Tay-Sachs Disease, what is their chance of having another child with Tay-Sachs Disease?
    - ☐ The same with each child born
    - ☐ Less with each child born
    - ☐ More with each child born
    - ☐ Unsure
23. What is the chance of being a carrier of the faulty gene for genetic conditions like Tay-Sachs Disease if you are Ashkenazi Jewish, compared with the general community?
    - ☐ The same
    - ☐ Higher
    - ☐ Lower
    - ☐ Unsure
24. Unlike the tests being offered through the school testing program, other genetic tests are available that may have implications for your own health. How important are these tests for *you*?
    - ☐ Not at all
    - ☐ Somewhat
    - ☐ Moderately
    - ☐ Very

ATTITUDES TOWARDS CARRIER GENETIC TESTING	AGREE	DISAGREE	UNSURE

25. These questions refer to the result you received from the school genetic carrier screening program. Now that you have the result of your genetic carrier test:

a. You will tell your partner when you are in a relationship	①	②	③
b. You will tell your family	①	②	③
c. You are ***likely*** to contact a genetic service to discuss your result soon	①	②	③
d. You are ***likely*** to contact a genetic service to discuss your result when you are planning to have children	①	②	③
b. You feel unhealthy	①	②	③
c. You feel worried about your own health	①	②	③
d. You feel worried about the health of your children	①	②	③
e. You feel angry	①	②	③
f. You feel scared	①	②	③
g. You feel depressed	①	②	③
h. You think you feel more unhealthy than your peers	①	②	③
i. You think you feel more worried than your peers	①	②	③

CHOICES ABOUT GENETIC TESTING	AGREE	DISAGREE	UNSURE
26. Everyone should be able to have genetic carrier testing for every condition that is available even if the test is not relevant to them based on their ancestry	①	②	③
27. High school is a good time for genetic carrier testing to be offered	①	②	③
28. If both partners in a couple carry the faulty gene for a condition, testing during pregnancy should be offered	①	②	③

29. How do *you* feel about new discoveries in genetics?

Please select the *most appropriate* response for *you* to these 6 statements by circling the number for each scale:

Bored	1	2	3	4	5	6	7	Excited
Valuable	1	2	3	4	5	6	7	Worthless
Uninterested	1	2	3	4	5	6	7	Interested
Indifferent	1	2	3	4	5	6	7	Passionate
Important	1	2	3	4	5	6	7	Unimportant

30. Do you have any suggestions for how you think the education session you participated in a year ago could be improved?

____

____

____

31. Do you have any other comments you would like to make?

____

____

____

____

YOU HAVE NOW COMPLETED THE SURVEY. THANK YOU FOR PARTICIPATING IN THIS RESEARCH.
